# Diversity of Acupuncture Point Selections According to the Acupuncture Styles and Their Relations to Theoretical Elements in Traditional Asian Medicine: A Data-Mining-Based Literature Study

**DOI:** 10.3390/jcm10102059

**Published:** 2021-05-11

**Authors:** Dong-Yeop Jang, Ki-Chang Oh, Eun-Seo Jung, Soo-Jin Cho, Ji-Yun Lee, Yeon-Jae Lee, Chang-Eop Kim, In-Jun Yang

**Affiliations:** 1 Department of Physiology, College of Korean Medicine, Gachon University, Seongnam 13120, Korea; ggg5438@gachon.ac.kr; 2 College of Korean Medicine, Dongguk University, Gyeongju 38066, Korea; okc77771@gmail.com (K.-C.O.); junges920@naver.com (E.-S.J.); chosujin936@naver.com (S.-J.C.); kei02127@naver.com (J.-Y.L.); yeonjae279@gmail.com (Y.-J.L.); 3 Department of Physiology, College of Korean Medicine, Dongguk University, Gyeongju 38066, Korea

**Keywords:** acupuncture, acupuncture points, acupuncture style, text mining, traditional Korean medicine, traditional Chinese medicine, *Dongeuibogam*, *Saamdoinchimgooyogyeol*, *Chimgoogyeongheombang*

## Abstract

Acupuncture point (AP) selections can vary depending on clinicians’ acupuncture style, and therefore, acupuncture style is an important factor in determining the efficacy of acupuncture treatment. However, few studies have examined the differences in AP selections according to the acupuncture styles and theoretical backgrounds causing the differences. We compared the AP prescriptions used for 14 diseases in three classical medical textbooks, *Dongeuibogam* (DEBG), *Saamdoinchimgooyogyeol* (SADI), and *Chimgoogyeongheombang* (CGGHB), which represent unique acupuncture styles and have affected clinicians during this time. AP prescriptions showed more diversity between textbooks than between types of diseases. Among the three textbooks, AP prescriptions of SADI were most different compared to those of DEBG and CGGHB. Importantly, we found each style can be more clearly explained by AP attributes than by the APs per se. Specifically, SADI, DEBG, and CGGHB preferred *five transport points* located on the limbs, APs of the *extra meridians*, and *source points*, respectively. This suggests the possibility that the theoretical diversity of acupuncture styles results in the heterogeneity of AP selections.

## 1. Introduction

Acupuncture has traditionally been used to manage various diseases, including low back pain [1,2], menopausal hot flashes [3,4], and poststroke rehabilitation [5,6]. In recent years, as people increasingly choose acupuncture to care for their health and treat diseases, the number of acupuncturists is also increasing [7].

As with other treatments, acupuncture point (AP) selections (also called AP prescriptions) differ according to the type of disease. Some AP selections are frequently used or recommended for certain diseases [8,9]. For example, SP6, HT6, KI7, KI6, CV4, LU7, LI4, and LR3 are commonly used for postmenopausal hot flushes [8]. BL24, BL25, BL31, BL33, BL31, and BL34 are frequently used for low back pain [10], among others. Simultaneously, AP selections can vary even for the same type of disease [11,12,13]. A previous study compared virtual AP selections prescribed by 80 clinicians and analyzed the variation of acupuncture, revealing the commonality and specificity of AP selections [14]. There are different types of acupuncture styles [15,16] that can make the difference between AP selections [17,18]. Moreover, differences in AP selections depending on the clinicians’ acupuncture styles can make substantial differences in treatment efficacy [17,18,19]. Therefore, clinicians’ acupuncture styles are an important factor in determining the AP selections and efficacy of acupuncture treatment. Indeed, when reporting the effects of acupuncture interventions in clinical trials, it is recommended to report clinicians’ acupuncture styles and AP selection together [20].

There are diverse theoretical elements in APs in traditional acupuncture theories, such as local or distant effects of points, five transport points, source points, and eight meeting points, etc. Each element presumes different aspects of *qi* (氣), *viscera and bowels* (臟腑, also called *ZangFu*), and *meridians* (經絡) [21]. This diversity of theories could affect the variation of acupuncture styles in practice. A study revealed that clinicians preferred some characteristics of APs for primary dysmenorrhea, suggesting that theoretical aspects affect acupuncture practice [22]. Despite the potential impact on clinical practice and research, few studies quantitatively examined the differences in AP selections according to clinicians’ acupuncture styles or investigated the underlying theory causing these differences in AP selections. 

In this study, we investigated how AP selections differed according to the clinician’s style and explained the differences in terms of theoretical elements. Because modern acupuncturists select APs by combining several acupuncture styles [16], it is difficult to study the differences and origins of AP selections according to acupuncture style by analyzing their AP prescriptions. Hence, we analyzed three classical medical textbooks, which mainly affect current traditional Korean medicine (TKM) practice of acupuncture: *Dongeuibogam* (DEBG, 東醫寶鑑, “Treasured Mirror of Eastern Medicine”), *Saamdoinchimgooyogyeol* (SADI, 舍岩道人針灸要訣, “Essential Rhymes on Acupuncture and Moxibustion by Master Saam”), and *Chimgoogyeongheombang* (CGGHB, 鍼灸經驗方, “Experiential Prescriptions of Acupuncture and Moxibustion”). Analyzing classical medical textbooks may provide the information needed to understand current acupuncture [23]. Our study aims to provide insight into the causes of AP selection diversity by comparing three classical medical textbooks. For quantitative analysis, we applied machine-learning-based methods, network analysis, and a nonparametric statistical test that was devised for this study.

## 2. Materials and Methods

### 2.1. Document Selection

**Figure 1:**
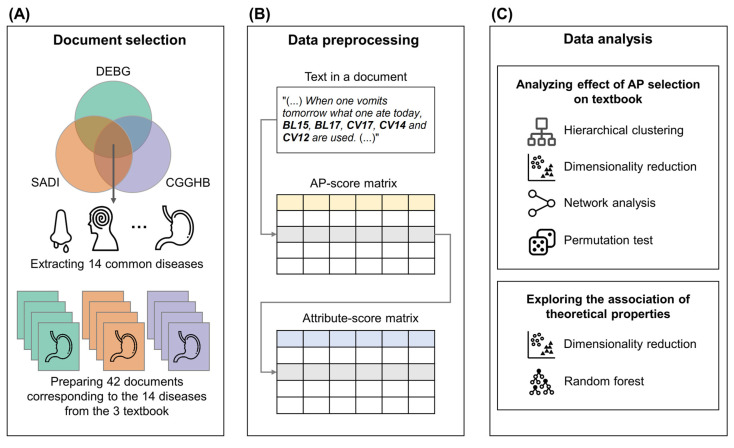
Study protocol. (**A**) Fourteen common diseases in three textbooks, DEBG, SADI, and CGGHB, were selected. For each textbook, 14 documents corresponding to the diseases were selected. (**B**) Texts, including AP prescriptions in each document, were transformed into AP-scores and AP attribute-scores. An AP-score matrix and an AP attribute-score matrix were constructed using the AP-scores and AP attribute-scores of the AP prescriptions. The rows of matrices represent the AP prescriptions, and the columns represent APs and attributes. (**C**) The effects of textbooks on AP prescriptions and their association with the theoretical attributes of APs were analyzed. AP: Acupuncture point, DEBG: *Dongeuibogam*, SADI: *Saamdoinchimgooyogyeol*, CGGHB: *Chimgoogyeongheombang*.

A document was defined as a chapter of DEBG, SADI, and CGGHB. Forty-two documents corresponding to the 14 diseases from the three classical medical textbooks were prepared: (1) aggregation (積聚), (2) vomiting (嘔吐), (3) cough (咳嗽), (4) eye disease (目病/目/眼), (5) ear disease (耳病/耳), (6) nasal disease (鼻病/鼻), (7) throat disease (喉症/咽喉), (8) dental disease (齒痛/齒/齒牙), (9) mouth disease (口病/口/口舌), (10) cholera (霍亂), (11) consumptive disease (虛損/虛勞), (12) head disease (頭痛/頭面/頭), (13) disease in lumbar vertebrae (腰痛/腰背/腰), and (14) abdominal disease (腹痛/腹脇/腹,脇) (Figure 1A). We selected diseases satisfying the following criteria: (i) diseases that were included in all three classical medical textbooks; (ii) diseases that were interpretable in modern medical perspectives. For example, internal damage (內傷) was excluded in our analysis due to the difficulty of interpreting it into the medical condition of modern medicine.

### 2.2. Data Preprocessing

To apply quantitative analysis to the documents, unstructured text data in documents need to be transformed into structured data. The acupuncture style used by clinicians is reflected in the AP element’s preference as well as AP prescriptions. From 42 documents, the texts that present AP prescriptions for each disease were extracted (Figure 1B). The extracted texts were preprocessed in two different ways: AP-scores and AP attribute-scores construction. An AP-score (or AP attribute-score) represents how much the APs (or AP attributes) are enriched in each document. The AP-scores (or AP attribute-score) are vectors of size (*n* × 1), where n is the number of APs (or AP attribute), and the attributes represent the relative frequencies of APs (or AP attributes) in each document. The AP-scores and AP attribute-scores were stacked horizontally, and an AP-score matrix and an AP attribute-score matrix were constructed, in which the rows represent documents. This study focused on four AP attributes: *distant points* (遠穴) points on *extra meridians* (奇經), *five transport points* (五輸穴), and *source points* (原穴). The points on the *extra meridians* mean APs of the *governor vessel* (督脈) and the *conception vessel* (任脈), which are not included in the *twelve meridians* (十二正經). *Eight extra meridians* (奇經八脈) were included in *extra meridians*, but only the *governor vessel* and the *conception vessel* have their own APs. *Distant points* mean the selected APs are far from the disease site. *Five transport points* mean five specific APs of the twelve meridians located distal to the elbows and knees, i.e., *well point* (井穴), *brook point* (滎穴), *stream point* (輸穴), *river point* (經穴), and *sea point* (合穴). The *source points* mean APs where the *original qi* (原氣) of the visceral organs pours, passes, or stays.

### 2.3. Similarity Analysis between AP Prescriptions

The similarities between AP prescriptions were compared to analyze the effects of the clinicians’ acupuncture styles on AP prescriptions. This study hypothesized that if similar AP prescriptions were used for other types of disease in the same classic medicine textbook, the AP prescriptions were influenced more by the clinician’s acupuncture style than the type of disease. Conversely, if similar AP prescriptions were used for the same disease in three different classic medicine textbooks, it was assumed that the AP prescriptions were influenced more by the type of disease than by the clinician’s acupuncture style. The following qualitative and quantitative methods were applied complementarily (Figure 1C).

#### 2.3.1. Hierarchical Clustering

The AP prescriptions were grouped according to their similarity by hierarchical clustering using the AP-score matrix. In this paper, the Euclidean distance between the AP-scores was calculated to determine the similarities of the AP-scores and AP attribute-scores; when the Euclidean distance between the AP-scores of documents was low, the similarity of the AP prescriptions was high.

#### 2.3.2. Dimensionality Reduction and Visualization

The differences between AP prescriptions were visualized by multidimensional scaling (MDS) using the AP-score and AP attribute-score matrices. The MDS visualizes the level of similarity of samples by translating the pairwise distances by projecting data onto two dimensions [24].

#### 2.3.3. Network Analysis

Network analysis is a process of investigating the structure of a network through the use of networks and graph theory. The topology of AP prescriptions’ networks was analyzed by constructing a network of documents based on the similarities between the AP-scores. The nodes and edges of the network were defined as documents and whether a couple of documents are similar, respectively.

#### 2.3.4. Permutation Test

The effects of textbooks and the effects of diseases were evaluated quantitatively by calculating the statistical significance of the mean of similarities between AP-scores in the null hypothesis. The null distribution was estimated by permuting the labels of original textbooks or diseases and computing the distribution of test statistics empirically under the null hypothesis. All null distributions were estimated from 100 million random samples. The empirical *p*-value was calculated from the null distribution.

#### 2.3.5. Contribution Score Calculation by Random Forest Classifiers

To determine which theory affects the AP selection for each textbook, random forest classifiers were trained to classify the original textbooks by AP attribute-scores, and feature importance was analyzed. In this paper, the feature importance of a random forest classifier is called the contribution scores of the attributes. A Wilcoxon signed-rank test was performed for each attribute in the AP attribute-scores.

### 2.4. Software

All methods were processed using Python (v. 3.6). Specifically, preprocessing was performed by Numpy (v. 1.17) and Pandas (v. 0.24.2). Data analysis was performed by Sklearn (v. 0.23) and visualized by Seaborn (0.11.0) and Matplotlib (v. 3.3.1). Network analysis was performed by Cytoscape (v. 3.6.0).

## 3. Results

### 3.1. AP Prescriptions Similarity in DEBG, SADI, and CGGHB

**Figure 2:**
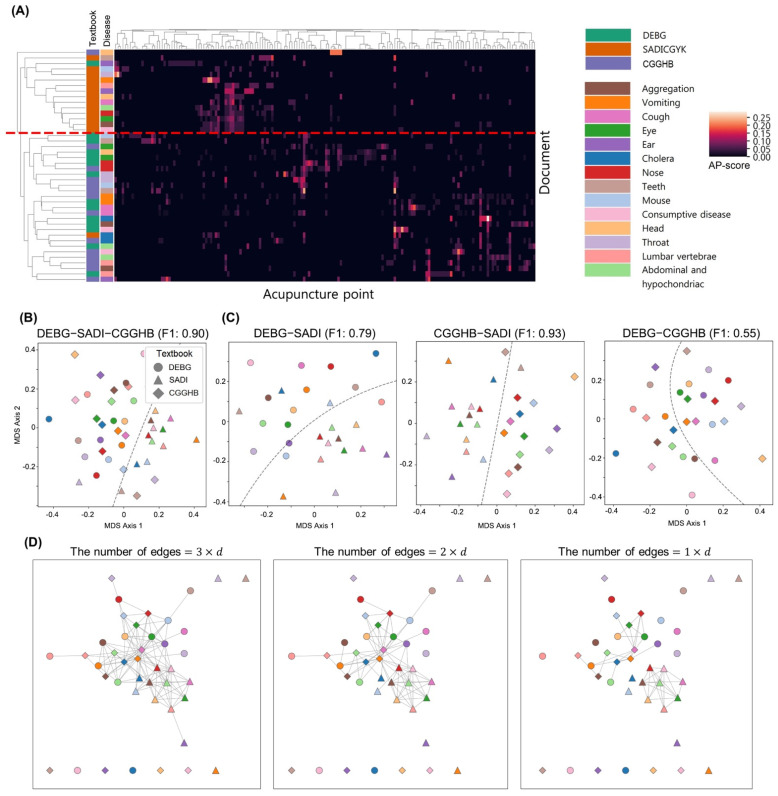
Visualization of the similarities of the AP-scores and their relationships with diseases and original textbooks. (**A**) Hierarchical clustering of the AP-score matrix. Each row represents a document. The left two columns indicate diseases and textbooks. The columns except for the two columns represent the APs. Note that the AP-scores are clustered by textbooks rather than diseases, as shown by a red dashed line. (**B**) Multidimensional scaling of the AP-scores from DEBG, SADI, and CGGHB. A dotted line shows a decision boundary in that the AP-scores that originated in SADI are separated from those that originated in the other textbooks. Indication of colors are the same as in Figure 2A. All decision boundaries obtained by the figures were calculated by applying support vector machines (kernel = radial basis function, C = 10, and gamma = 1). (**C**) Multidimensional scaling of the AP-scores from pairs of textbooks, (DEBG, SADI), (SADI, CGGHB), and (DEBG, CGGHB). The dotted lines show the decision boundaries that divide the original textbooks. Indication of colors are the same as in Figure 2A. F1 indicates the F1 score of the decision boundary. Note that the AP-scores of SADI are distinguished from those of other textbooks, while the AP-scores of DEBG and CGGHB are not distinguished from those of each other. (**D**) Networks of AP-scores. To avoid bias from arbitrary thresholding, the networks were visualized with different sparsity levels. Indication of colors are the same as in Figure 2A. *d* indicates the number of AP-scores (*d* = 42). AP: Acupuncture point, DEBG: *Dongeuibogam*, SADI: *Saamdoinchimgooyogyeol*, CGGHB: *Chimgoogyeongheombang*.

The effects of types of disease and clinicians’ acupuncture styles on the AP-scores were analyzed by hierarchical clustering, MDS, and network visualization. Hierarchical clustering was conducted to group the AP-scores based on their similarities. Figure 2A shows that the AP-scores derived in SADI are strongly clustered, suggesting that the AP prescriptions of SADI are highly homogeneous. MDS was conducted to determine if the AP prescriptions are divided by clinicians’ acupuncture styles. Figure 2B,C shows the AP-scores divided by SADI and the other classical medical textbooks. However, The AP-scores of DEBG and CGGHB were clearly distinguished by the type of disease, rather than textbooks (Figure 2C). This suggests that the acupuncture styles used by DEBG and CGGHB could be similar.

Network visualization was conducted to analyze the pairwise similarities of AP prescriptions and the topology of the AP prescriptions. Figure 2D shows that the edges between nodes corresponding to SADI are retained in the network with a strict threshold, whereas the edges between nodes corresponding to the same diseases are not. This confirms that the AP prescriptions of SADI are homogeneous and have an analogous pattern that can be applied to any disease.

### 3.2. Quantitative Analysis of Similarity of AP Prescriptions in DEBG, SADI, and CGGHB

Next, empirical *p*-values using permutation tests were conducted to determine the significance of the clinicians’ acupuncture style and type of disease. The effects of the clinicians’ acupuncture style and type of diseases on AP variations were significant (*p* < 10^−8^ and *p* = 0.012, respectively) (Table 1). This suggests that AP prescriptions reflect the difference between the type of disease and the acupuncture styles of clinicians. In particular, the effect of SADI was greater than random in the other tests with DEBG or CGGHB (*p* = 3.2 × 10^−7^ and <10^−8^, respectively) (Table 1). The AP prescription used in SADI is unique compared to the other classical medical textbooks, suggesting that the unique acupuncture style used in SADI made this difference significant.

The effects of the type of diseases on AP prescriptions were statistically significant in the permutation test with DEBG and CGGHB (*p* = 1.8 × 10^−7^), and the effects of the clinicians’ acupuncture styles on AP prescriptions were not significant but showed trends to some degree (*p* = 0.066) (Table 1). These results suggest that the acupuncture styles used in DEBG and CGGHB have their style, but there were some similarities between these two acupuncture styles compared to the SADI. Unlike the SADI acupuncture style, the DEBG and CGGHB acupuncture styles employed unique APs according to the disease type. The quantitative results support the results of visualization analysis in that AP prescription was significantly affected by the type of disease and the clinicians’ acupuncture styles.

### 3.3. AP Attribute Preference in DEBG, SADI, and CGGHB

**Figure 3:**
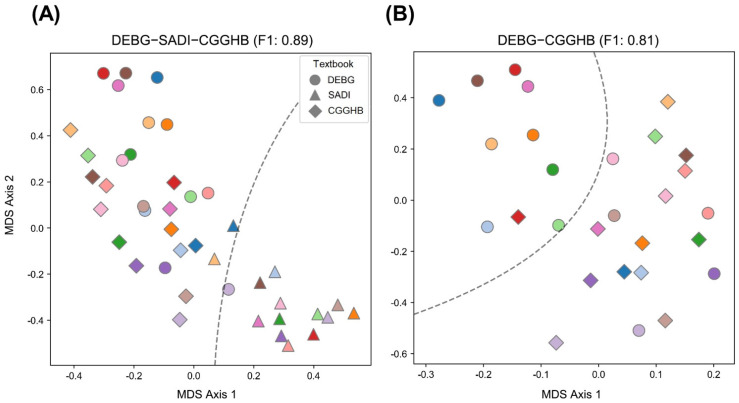
Visualization of the similarity of the AP attribute-scores and their relationship with the original textbooks and diseases. (**A**) Multidimensional scaling of the AP attribute-scores originated from DEBG, SADI, and CGGHB. Indication of colors are the same as in Figure 2A. The dotted line shows a decision boundary that divides the SADI and the others. (**B**) Multidimensional scaling of the AP attribute-scores originated from DEBG and CGGHB except for SADI. Indication of colors are the same as in Figure 2A. A dotted line shows a decision boundary that divides DEBG and CGGHB. F1 indicates the F1 score of the decision boundary. Note that the decision boundary moderately divides the AP attribute-scores that originated in DEBG from those that originated in CGGHB. All decision boundaries obtained by the figures were computed by applying support vector machines (kernel = radial basis function, C = 10, and gamma = 1). DEBG: *Dongeuibogam*, SADI: *Saamdoinchimgooyogyeol*, CGGHB: *Chimgoogyeongheombang*.

The acupuncture style used by clinicians is reflected in the selection of AP attributes. [22] MDS was performed on the AP attribute-scores of all AP prescriptions in DEBG, SADI, and CGGHB. As shown in Figure 3A, the AP prescriptions of SADI were separated, even when using the AP attribute-scores. MDS with the AP attribute-scores corresponding to the AP prescriptions originating in DEBG and CGGHB, except for SADI, was performed to determine the differences between DEBG and CGGHB. Surprisingly, in DEBG and CGGHB, which did not show any difference when divided by APs per se, a distinction was noted when divided by the AP attributes. (Figure 3B) This result contrasts with a previous analysis in that the AP-scores of DEBG and CGGHB were not divided. This suggests that the variation of AP prescriptions between DEBG and CGGHB can be more clearly explained by AP attributes rather than by the APs per se.

**Figure 4:**
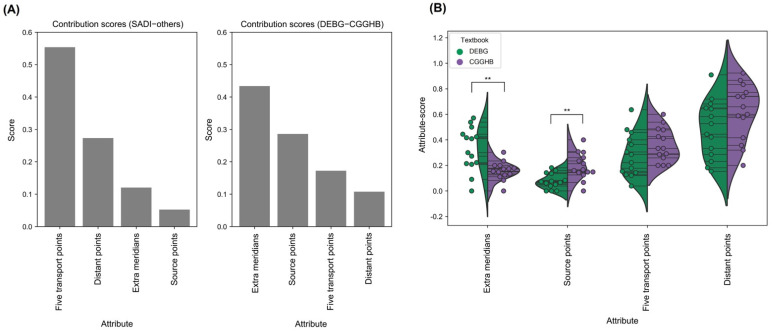
Contributions of the attributes to the difference between textbooks. (**A**) Contribution scores of the attributes from random forest models. Each random forest model was trained to distinguish between SADI and others (left) or DEBG and CGGHB (right). (**B**) Comparison of the distributions of AP attribute-scores between DEBG and CGGHB for each AP attribute. Each point represents an AP attribute-score of the document. The attributes were ordered by the contribution scores of the random forest classifier. DEBG: *Dongeuibogam*, SADI: *Saamdoinchimgooyogyeol*, CGGHB: *Chimgoogyeongheombang*. ** Benjamini–Hochberg corrected *p*-value < 0.01.

To determine which AP attribute is preferred in each textbook, random forest classifiers were trained to predict a reference textbook of AP prescriptions with its AP attribute-scores, and the trained classifiers were analyzed. As shown in Figure 4A, five transport points were the most crucial feature to classify AP prescriptions of SADI and others. In contrast, *extra meridians* and *source points* were important features to classify the AP prescriptions of DEBG and CGGHB. In particular, Figure 4B reveals that the points on *extra meridians* are enriched significantly in DEBG, whereas the *source points* are enriched significantly in CGGHB (*p* < 0.01, respectively). This suggests that each acupuncture style has its own preferred AP attribute; SADI prefers *five transport points* located on the limbs, DEBG performs *extra meridians*, and CGGHB uses *source points*. This shows that the preferred AP attributes may vary according to the clinician’s acupuncture style and background theories.

## 4. Discussion

A wide variety of AP selections are currently in use [23,25]. The Standards for Reporting Interventions in Clinical Trials of Acupuncture (STRICTA) guidelines recommend reporting the acupuncturist’s educational background when reporting the efficacy of acupuncture treatment [20]. In particular, treatment efficacy and physical effects may vary depending on AP prescriptions [17,18,19,26,27,28]. Hence, the diversity of acupuncture selections needs to be considered to improve the efficacy and clinical research quality of acupuncture treatment. Owing to the great diversity among AP selections, it is difficult to derive the optimal AP selections or to explain the rationale for AP selection [22]. Few studies have examined the differences in AP selection between clinicians and theoretical differences contributed to the diversity of AP selections. Here, we quantitatively analyzed the differences in AP prescriptions used for 14 diseases recorded in DEBG, SADI, and CGGHB and tried to explain the differences in terms of the theoretical elements in traditional Asian medicine (TAM). We found that AP selections varied according to styles, and this diversity is associated with differences in the preference of theoretical elements.

In this study, we analyzed AP prescriptions in the Korean classical medical textbooks, rather than those from patients’ records. Even in modern acupuncture practice, clinicians’ acupuncture style is largely influenced by the theoretical background of TAM. Hence, comparing the prescriptions from classical medical textbooks is an effective strategy for investigating the differences in AP prescriptions or theoretical preference according to the acupuncture style.

Our results showed that even for the same disease, the AP prescriptions used in the three classical medical textbooks were remarkably different. For the example of treatment of cough, 18 APs were selected in DEBG, of which BL13 was selected six times and was the most used. BL13 was used four times in CGGHB but was not selected in SADI. In CGGHB, 31 APs were selected, and LI4 was selected six times, making it the most used. However, LI4 has never been used in both DEBG and SADI. Fourteen APs were selected in SADI, of which SP3 was selected three times and was the most used. However, this AP is not used in both DEBG and CGGHB.

It is known that SADI was written based on DEBG and CGGHB, but the AP prescription recorded in SADI was the most different compared to the other two classical medical textbooks. The homogeneity between the AP selections used in SADI was high, even for the case in which the type of disease was different. In SADI, SP3 is the most frequently selected in both aggregation, cough, eye disease, nasal disease, head disease, consumptive disease, and disease in lumbar vertebrae, but it has never been used in DEBG and CGGHB.

When clinicians choose APs, they usually consider these AP attributes: whether the AP is local or distant from diseased sites, whether the AP can be specifically used in the disease, which meridians the AP is contained in, and how much manipulating the AP is painful [29]. We consider that these attributes reflect traditional theories in acupuncture practice. Interestingly, we found that the AP attribute preference can explain the difference in textbooks even when the APs could not. In detail, the effects of styles of DEBG and CGGHB on APs per se were not significant. However, DEBG used more APs belonging to the *governor vessel* and the *conception vessel* statistically significantly; meanwhile, CGGHB used more APs belonging to *source points*. These results indicate the possibility that the different patterns in the AP selections between DEBG and CGGHB are caused by the underlying theoretical background in each style.

The *governor vessel* and *conception vessel*, which belong to the *eight extra meridians*, are known to be directed to all of the *yang and yin channels* (陰陽經) and regulate the *body qi* circulation [30]. The theoretical background of an AP selection in DEBG, which emphasizes *systemic qi* circulation and yin-yang balancing, is influenced by the Taoism theory that disease is not caused by a single etiology but by complex physical, mental, and social interactions [31]. The conception vessel APs may be effective in preventing and treating diabetes identified by a yin deficiency [30]. Diseases related to the *governor vessel* are involved with respiration, digestion, and the urinary reproduction organ [32]. A recent study reported that stimulating APs belonging to the *governor vessel* and the *conception vessel* could stimulate the parasympathetic division of the visceral motor system, which could promote visceral movement [33].

A feature found in AP selections of CGGHB is that it uses many *source points*. The *source points* are located primarily on the pulsation point of the wrist and ankle [33]. It has been reported that the source point has lower electrical resistance and higher electrical potential than other sites [34,35]. Regarding the locational characteristics of the *source points*, *source points* were used mainly to treat cardiovascular diseases [35]. According to traditional medical theory, it is possible to diagnose and treat diseases of the 12 meridians and intestines through *source points* [35]. A clinical study reported that the *source points* could react sensitively with organs, such as the liver and intestines, and produce better therapeutic effects [36]. The use of *source points* means to treat the organs and meridians that are the root causes of the disease. This therapeutic strategy appears to be a different theory from modern acupuncture using local APs. 

The acupuncture selection recorded in SADI consists mainly of *five transport points*. According to traditional theory, stimulating the *five transport points* can facilitate the movement of the *meridian qi* to which the APs belong and even regulate the role of viscera and bowels corresponding to the meridian [37]. The *five transport points* theory originated from the *five phases* (五行, also called five elements) theory in an ancient Chinese classical textbook called *Nanjing* (難經), but the acupuncture prescriptions recorded in SADI contain a unique theory developed by the Korean Buddhist monk known as *Saam* (舍巖) [38]. This differed from Chinese acupuncture methods in that it used the *five transport points* belonging to the self-meridian and other meridians together according to the pattern identification [39]. Hence, the therapeutic aim of SADI acupuncture prescriptions is not only to control the balance among the *five phases* but also to control the *Cold-Heat and Deficiency-Excess Pattern* (寒熱虛實) balance between *viscera and bowels*. According to recent studies, stimulating the *five transport points* could increase parasympathetic nerve activation and regulate the balance of the autonomic nervous system. Therefore, it has the advantage of producing systemic effects in addition to the local effects of acupuncture [38,39]. The *five transport points* occupy a large area in the cortical representation of the postcentral sensory gyrus in the brain, so stimulating these APs could trigger a greater response in the brain [40,41]. Because the *five transport points* are located at the ends of the four limbs, stimulation of these APs has the advantage of safely performing acupuncture without risking damage to the internal organs.

Our study has some limitations. We did not consider the pattern identification of the AP selection in this study. In TAM, a diagnostic method called pattern identification is used to synthesize the patient’s symptoms and make treatment decisions. Even for the same disease, pattern identification is varied, and the diversity of pattern identification results in the heterogeneity of AP selections [42,43]. Therefore, for a more comprehensive understanding of the heterogeneity of AP selections, it is needed to analyze how pattern identification differs according to the clinician’s acupuncture style and how this contributes to the diversity of AP selections. Reviewing the classical medical textbooks can provide meaningful information to understand current AP prescriptions [23]. Despite the fact that classical medical textbooks have affected current AP prescriptions, however, they are not the same [25,44]. Further research based on data obtained from clinicians with different acupuncture styles is necessary to confirm our literature analysis-based results. The acupuncture styles and diseases we analyzed are limited to only 3 styles of TKM and 14 diseases. In particular, AP selections we analyzed are all from traditional Korean medicine textbooks. We believe, however, that our findings of the diversity in the AP selections and their relations to the theoretical elements are not limited to TKM, given that the traditional medicine in East Asian countries shares lots of commonality in a broad sense. It will be valuable to extend our approach to broader styles in TAM and more various diseases in future studies.

## 5. Conclusions

Acupuncture style has a significant impact on AP selection, and the impact can be explained by the theoretical elements underlying each style. This suggests the possibility that the theoretical diversity of acupuncturists results in the heterogeneity of AP selections. These findings could be used in the design of future acupuncture clinical studies.

## Figures and Tables

**Table 1:** Similarities between the AP-scores in the same textbook and the same disease. The Euclidean distance was used to determine the similarities: when the Euclidean distance between the AP-scores of documents was low, the similarity of the AP prescriptions was high. DEBG: *Dongeuibogam*, SADI: *Saamdoinchimgooyogyeol*, CGGHB: *Chimgoogyeongheombang*.

Textbooks	Type	Observed Similarity	Expected Similarity	Empirical *p*-Value
DEBG, SADI, and CGGHB	Same textbook	0.3558	0.3751	<10^−8^ ***
	Same disease	0.3628	0.3751	0.012 *
DEBG and SADI	Same textbook	0.3541	0.3722	3.2 × 10^−7^ ***
	Same disease	0.3832	0.3722	0.89
SADI and CGGHB	Same textbook	0.3449	0.3680	<10^−8^ ***
	Same disease	0.3789	0.3680	0.85
DEBG and CGGHB	Same textbook	0.3685	0.3711	0.066 ^#^
	Same disease	0.3264	0.3711	1.8 × 10^−7^ ***

^#^ Benjamini–Hochberg corrected *p*-value < 0.1; * Benjamini–Hochberg corrected *p*-value < 0.05; *** Benjamini–Hochberg corrected *p*-value < 0.001.

## Data Availability

The data presented in this study are available on request from the corresponding author.
